# Excessive smartphone use and its correlations with social anxiety and quality of life among medical students in a public university in Malaysia: A cross-sectional study

**DOI:** 10.3389/fpsyt.2022.956168

**Published:** 2022-11-24

**Authors:** Hajar Mohd Salleh Sahimi, Mohd Hafiz Norzan, Nik Ruzyanei Nik Jaafar, Shalisah Sharip, Ammar Ashraf, Kamaleshini Shanmugam, Nur Shahirah Bistamam, Najat Emir Mohammad Arrif, Saathish Kumar, Marhani Midin

**Affiliations:** ^1^Department of Psychiatry, Faculty of Medicine, Universiti Kebangsaan Malaysia, Cheras, Malaysia; ^2^Department of Psychiatry, Kuala Lumpur Hospital, Kuala Lumpur, Malaysia

**Keywords:** social phobia, medical student, smartphone dependence, quality of life, self esteem

## Abstract

**Introduction:**

Smartphone usage has significantly increased in the last decade among young adults has significantly increased in the last decade. While its benefits are undeniable, its negative implications are increasingly emerging. Studies are needed to investigate the effects of excessive smartphone use on a young person's life. This study aimed to determine the prevalence of excessive smartphone use among medical students and its relations with social anxiety, self-esteem, and quality of life.

**Methods:**

A cross-sectional study was conducted among medical students from Universiti Kebangsaan Malaysia (UKM) in UKM Medical Center. A total of 273 students have consented to participate and completed self-reported questionnaires encompassing sociodemographic information, the Short Version Smartphone Addiction Scale (SAS-SV), the Social Interaction Anxiety Scale (SIAS), the World Health Organization Quality of Life (WHOQOL-BREF) and the Rosenberg Self-esteem Scale (RSES). Sociodemographic data, SIAS score, WHOQOL-BREF score and the Rosenberg Self-esteem Scale score were treated as independent variables. Smartphone addiction Scale score was treated as the dependent variable. Bivariate analysis was used to explore the relationship between independent and dependent variables using the Fisher exact test, Pearson Chi-Square and Pearson correlation coefficient. Multiple linear regression analysis was used to analyze the variables with a *p*-value of < 0.05 from the Pearson correlation coefficient test.

**Results:**

The percentage of excessive smarphone use among UKM medical students is 48%. The bivariate analysis showed that excessive smartphone use has a small but significant positive correlation with social anxiety (*r* = 0.173, *p* = 0.004) and negative correlations with physical health (*r* = −0.133, *p* = 0.028), psychological wellbeing (*r* =−0.135, *p* = 0.026), social relationships (*r* = −0.232, *p* = 0.001), environment (*r* = −0.260, *p* = 0.001) and self-esteem (*r* = −0.128, *p* = 0.035). In the multiple regression analysis, a better environment predicted a reduced risk for smartphone addiction (β = −0.233, *p* = 0.013).

**Conclusion:**

Almost half of the students were found to have smartphone overdependence. Excessive smartphone use has shown a significant relationship with an increased risk for social anxiety, reduction in self-esteem, and quality of life among medical students. A closer look into the possible intervention is needed in the future to curb the negative effects arising from excessive smartphone use.

## Introduction

The usage of smartphones has significantly increased during the last decade, particularly among young adults. In Malaysia, smartphone usage in 2021 was approximately 98.7%, a slight increase from 98.2% in 2020 (1). If utilized correctly, smartphones offer many important functions that extend beyond their traditional purpose as communication devices. These functions act as mediums to enhance one's quality of life (2) and help to boost individual self-improvement (such as minimizing depressive symptoms) (3). In the clinical setting, smartphone-based interventions such as online psychotherapy have effectively treated psychological conditions (such as depression), improved quality of life, and reduced stress levels (4). Other than that, certain smartphone apps may help to save lives during emergencies. Several such examples are apps that offer guides on cardiopulmonary resuscitation (CPR) for the general public (5), diagnose skin cancer (6), detect road traffic accidents, or provide smart rescue systems (7).

Nonetheless, with smartphones' growing necessity in our daily activities, social and psychological problems have risen due to their overuse. A clear obstacle to a systematic investigation into this issue is the lack of a clear definition of a “smartphone addiction”, and the unavailability of its diagnosis in the International classification of disease 11 (ICD-11) (8) or Diagnostic and Statistical Manual 5 (DSM-5) (9). Most studies have assumed that it is an addictive behavior, even though there is still active discussion on whether excessive smartphone use is part of a continuum of addictive behaviors (10). To most researchers, excessive smartphone use has been considered a form of technological addiction (11), which has been defined as a behavioral addiction that involves interaction between a human and a machine that is non-chemical (12, 13). This present study also utilizes the same concept – that is, smartphone addiction is a type of behavioral addiction – when constructing its smartphone measurements. Furthermore, a study in the past has shown that smartphone addiction exhibits several similar aspects to substance-related dependence including a) compulsive behavior, b) withdrawal, c) tolerance, and d) functional impairment (11). Besides that, the prevalence of smartphone addiction varies from country to country where past research has found the levels to be 26.6% in Korea (14), 26.8% in India (15), 9.3% in Tehran (16), and 16.9% in Switzerland (17). In Malaysia, a recent study found a much higher prevalence rate of 40.6% (18).

For this paper, the term “excessive smartphone use” (ESU) will be used due to the lack of a clear and established clinical diagnostic definition. ESU is a complex and multifactorial condition. A theory that explains this condition is the “Object Attachment Theory” (19) which originated from the Attachment Theory by Bowlby (20). Object Attachment Theory describes the bonding relationship between humans and inanimate objects such as smartphones. Individuals who are attached to their smartphones perceive the object as a surrogate for comfort and security (19). Losing access to smartphones might then cause intense discomfort or nomophobia for these individuals (21). Attachment to smartphones is not merely driven by the need to connect with other people (22). Instead, individuals might be using their smartphones for other non-social purposes such as watching movies, playing games, or reading the news. Smartphones are easily accessible and portable and are not only used for online internet consumption, but also offline activities such as telecommunication (phone calls), taking pictures, or playing games. In contrast, internet addiction is more specific to problematic compulsive use and consumption of content on the internet. Therefore, smartphone use is different from traditional internet use *via* desktop computers, due to the former's ability to be used anywhere and at any time (23). In addition, constantly checking smartphones and feeling fearful when not holding one are among the other distinguishing factors between the two addictions mentioned above. For this reason, the scope of this current study shall be limited to investigating “excessive smartphone use” instead of “internet addiction”.

Various studies have found that ESU correlates with impaired psychological wellbeing, such as depression, anxiety, and stress (15, 24). Some studies conducted within the Malaysian context have arrived at the same conclusion as well (18, 25). Furthermore, ESU is associated with other psychological conditions such as obsessive-compulsive disorder (OCD) (26), attention deficit hyperactivity disorder (ADHD) (27), and insomnia (28). Moreover, more and more recent research has demonstrated a higher relationship between social anxiety and ESU (29, 30).

Social anxiety or social phobia is a condition characterized by marked fear of being scrutinized or humiliated by others (31). Social anxiety causes difficulties in in-person communication with others. Whether individuals with social anxiety use mobile devices more than others or vice versa, remains a question. Interactions using smartphones are relatively less anxiety-provoking than physical, face-to-face meetings. However, it may cause individuals to be more vulnerable to the excessive use of smartphones (29). Furthermore, a positive correlation has been found between anxiety and smartphone usage among university students (32). Specifically, a positive correlation has been most profound between ESU and social phobia (29), a finding that echoes the discovery made in another study (33).

Looking at the relationship between ESU and social anxiety in terms of social interaction, it seems that those who have ESU reported a higher association with loneliness and shyness relative to those who do not (34–36). Smartphones are regarded as important social devices, where *via* their usage, one may construct an extensive social network, build a self-image, and feel connected with the rest of the world (37). Nonetheless, research concerning the interaction model between social interaction and ESU is still in its infancy. Individuals with a fear of “real-life” social interaction may be more prone to using smartphones to communicate. Similarly, individuals experiencing ESU may be too occupied with their gadgets and consequently distance themselves from interacting with people in real life. A study showed that interaction anxiety significantly affects ESU (38). Meanwhile, another study demonstrated that social skills (social expressivity skills) predicted ESU and that the latter can be a mediating factor between social skills and psychological wellbeing (39). Moreover, social anxiety and reduced self-efficacy have also been shown to mediate ESU (38). Besides that, another study suggested that social anxiety plays a mediating role between poor self-esteem and ESU (40), while cognitive-emotional regulations mediated the relationship between social anxiety and ESU (41).

Smartphone use has generally been affecting an individual's quality of life. A healthy engagement in online activities may give pleasure in life and add to its overall quality. However, problematic use of these gadgets may lead to a neglect of other useful tasks and responsibilities that would inevitably impair a person's quality of life. According to the World Health Organization (WHO), quality of life consists of four domains: physical health; psychological wellbeing; social relationships; and the environment (42). Studies have shown that ESU is negatively correlated with the quality of life (43), while the former predicts the latter (44). Moreover, there is currently a debate about the interaction between ESU and self-esteem levels. One large-scale study conducted in Norway reported that the addictive use of social media is linked to low self-esteem (38, 45), a finding that has been replicated in several other studies (46, 47). Self-esteem was also revealed to be the mediator between the effect of social media addiction and life satisfaction (48), where a high level of self-esteem is found to be a protective factor against ESU (49). On top of that, looking at the available evidence, previous studies showed that social anxiety might lead to ESU (29, 50). In another study, mobile phone addiction might be mediated by poor self-esteem (40) and cognitive-emotional regulation (41), effectively leading to poor quality of life (42). However, further studies are still needed to confirm these findings.

Medical students are among those with a high rate of smartphone use (18). The students often use smartphones to obtain study materials, make notes, or search for answers for their assignments (51). This tendency to rely on smartphones might put them in the risk group for excessive use of the device. In Malaysia, studies on ESU are still lacking, which is not in line with the high rate of smartphone use observed in the nation. It is a concern that smartphone use, especially when it is excessive, may result in negative implications for young adults. Having these concerns as aspirations, this study aims to determine the rate of ESU and the potential risk factors associated with it among medical students at the Universiti Kebangsaan Malaysia Medical Center (UKMMC).

## Methods

This study is cross-sectional in design and involves 1st- and 4th-year medical students at UKMMC. Data collection was conducted using convenience sampling, where year 1 and 4 students who are present on campus during the data collection period were approached after classes. The year-1 students represent those undergoing pre-clinical rotations, while the year-4 students represent those undergoing clinical rotations. The sample size of this study was determined using a manual sample size calculation formula for the prevalence study (52) based on the method by Haug et al. (17), who conducted a similar study on students in Switzerland. The sample size calculation was per the following formula:


$$\begin{array}{l} \text{n }=\;{\left(\text{Z}1-\alpha \right)}^{2}\;\left[\text{P}\left(1-\text{P}\right)/\text{D}2\right] \end{array}$$

where,

Z1-α = Z0.95 = 1.96 (for a confidence interval of 95%, Z = 1.96; normal distribution table).

P (Prevalence) = 0.17, was taken from the prevalence of smartphone addiction in a study among students in Switzerland (17).

D (Absolute precision required) = 5% = 0.05.

Therefore, *n* = 1.962 [0.17(1–0.17)/0.052] = 217. The final estimation of the sample size required is estimated to be a minimum of 260 students (217 + 43 = 260, where 43 represents an a priori provision of 20% non-responders).

### Data collection

The data collection process was conducted between 15^th^ June 2018–30^th^ June 2018. Briefing on the purpose and nature of the study, as well as the inclusion and exclusion criteria, were delivered to the sampled students before the interview. The inclusion criteria were twofold: (1) the participant must be a year-1 or year-4 undergraduate medical student at UKM; and (2) the participant must consent to voluntarily take part in this research. The exclusion criterion was a participant who does not give his/her consent. Those who fulfilled the criteria were given a set of self-report questionnaires and written consent. The participants were asked to complete the questionnaires that consist of questions regarding their socio-demography, the short version of the Smartphone Addiction Scale (SAS-SV) (53), the Social Interaction Anxiety Scale (SIAS) (54), WHO Quality of Life-BREF (WHOQOL-BREF) (42), and Rosenberg Self-esteem Scale (RSES). The authors were present at each briefing to help and clarify any doubts about the questionnaires. All participants were assured of the study's confidentiality. The time needed to complete the questionnaires ranged from 20 to 30 min for each student. The students were informed that professional help is available if they need further consultation.

Written permission and approval to conduct the study were obtained from the Research and Ethics Committee, Faculty of Medicine, Universiti Kebangsaan Malaysia (Project Code: FF-2018-243).

### Study instruments

#### The sociodemographic questionnaire

This is a self-reported questionnaire that includes questions on respondents' age, ethnicity, religion, year of study, place of origin (urban/rural), parents' living status, parents' marital status, parents' household income, existing medical condition, and existing psychiatric condition.

#### The short version smartphone addiction scale

The first Smartphone Addiction Scale (SAS) questionnaire was developed and validated by Kwon et al. (55). The original version consisted of 33 questions with a 6-point Likert scale ranging from 1: strongly disagree and 6: strongly agree. All six questions represent six factors being measured. The factors are; 1) daily-life disturbance; 2) positive anticipation; 3) withdrawal; 4) cyberspace-oriented relationship; 5) overuse; and 6) tolerance. The internal consistency and concurrent validity of SAS were verified, with a Cronbach's alpha of 0.967.

A shorter version of SAS was created and validated again in the same year by Kwon et al. (53). The shorter version of the SAS (or SAS-SV) consists of only 10 questions. This version of the psychometric test showed good internal consistency and concurrent validity with Cronbach's alpha of 0.911. The SAS-SV was significantly correlated with the original version of SAS and other similar scales, such as the Smartphone Addiction Proneness Scale (SAPS) and The Korean Self-reporting Internet Addiction Short-form Scale (KS-Scale). The ROC analysis results showed an area under the curve (AUC) value of 0.963 (0.888–1.000), with a cut-off value of 31 for males and 33 for females. The same cut-off values were used in this study. Based on the above discussion, it may be said that the SAS-SV showed good reliability and validity for the assessment of smartphone addiction (53). A validation study of the Malay-translated version of the SAS-SV was conducted by Ching SM et al. among medical students in Malaysia. This version has also demonstrated good internal consistency and concurrent validity with a Cronbach's alpha value of 0.94 (56). We used the validated English version of the SAS-SV, as medical students in Malaysia generally have a good mastery of and proficiency in the English language. The Cronbach's alpha for our current study sample for SAS-SV is 0.86.

#### Social interaction anxiety scale

The Social Interaction Anxiety Scale (SIAS) is a self-report scale that measures the anxiety experienced by a person during social interactions with others. This scale was first developed and validated by Mattick and Clarke (54). The scale contains 20 items where the respondent rates how much each item relates to them using a 5-point Likert scale, ranging from 0 points (Not at all characteristic of me), 1 point (Slightly characteristic of me), 2 points (Moderately characteristic of me), 3 points (Very characteristic of me), 4 points (Extremely characteristic of me). The first validation study revealed that SIAS has a good internal consistency (Cronbach's α = 0.94) and test-retest reliability (Cronbach's α = 0.92) (54).

In terms of its discriminant validity, SIAS has been compared to other scales that measure social anxiety, such as the Social Phobia Scale (SPS) and the Social Phobia & Anxiety Inventory (SPAI) by Peters (57). The SIAS was significantly correlated with SPS & SPAI, suggesting that they have a similar construct. However, SIAS does not differentiate between social anxiety and other types of anxiety disorders (57). To interpret the SIAS scores, Peters defined the cut-off score as 36 for probable social anxiety with a sensitivity of 0.93, specificity of 0.60, a positive predictive value (PPV) of 0.84, and a negative predictive value (NPV) of 0.78 (57). To the best of our knowledge, there is no specific psychometric study of SIAS performed in the Malaysian setting. There were studies in Malaysia that utilized the SIAS questionnaire but the authors (58, 59) did not conduct any psychometric studies to complement the main study. The Cronbach's alpha for our current study sample for SIAS is 0.89.

#### WHOQOL-BREF

The WHO's definition of Quality of Life (QoL) is “An individual's perceptions of their position in life in the context of the culture and value systems in which they live and about their goals, expectations, standards, and concerns” (42). WHOQOL is a questionnaire developed by WHO to assess the level of QoL of a person. The shorter version of WHOQOL (or WHOQOL-BREF) was introduced to improve the original questionnaire's practicality and has been tested in 20 field centers across 18 countries. Moreover, the latter is now available in 19 languages (60).

The WHOQOL-BREF consists of 26 items (a significant reduction in comparison to the 100 items contained within the original WHOQOL). In total 24 of these items are divided into four domains: physical; psychological wellbeing; social relationship; and environmental. The remaining two items represent the person's perception of their overall QoL and general health. A 5-points Likert scale is used for the questionnaire's scoring. The total raw score for each of the four WHOQOL-BREF domains can be obtained by summing the item scores and converting them to a scale ranging from 0 to 100 using the formula below:


$$\begin{array}{l} \text{Transformed Scale }=\;(\text{Actual raw score}\\ \;-\text{ lowest possible raw score})\\ \;\times \;100\text{ possible raw score range} \end{array}$$

The original WHOQOL (U.S version) has an acceptable internal consistency with Cronbach's alpha of 0.82–0.95 across its domains (61). Meanwhile, the WHOQOL-BREF has a high correlation level with the WHOQOL-100, registering a Cronbach's alpha of 0.89 (62). According to the WHOQOL-BREF manual, higher scores indicate a higher QoL and vice versa. To ease interpretations, Hawthorne et al. set a reference point for the scores. Their results showed that the general levels for the WHOQOL-BREF domains were 73.5 (SD = 18.1) for the Physical Health domain, 70.6 (SD = 14.0) for the Psychological wellbeing domain, 71.5 (SD = 18.2) for the Social Relationships domain, and 75.1 (SD = 13.0) for the Environment domain (63). WHOQOL-BREF has been translated into Malay and validated in the local Malaysian setting in a psychometric study of the Malay Version-WHOQOL-BREF by Hasanah et al. This study showed that WHOQOL-BREF has a good internal consistency (Cronbach alpha of 0.89), good test-retest reliability (ICC of 0.75), good construct validity (based on Exploratory Factor Analysis), and good discriminant validity when applied to the local context (64). The Cronbach's alpha for our current study sample for WHOQOL-BREF is 0.92.

#### Rosenberg self-esteem scale

This is a self-rated scale developed by Rosenberg to measure the level of self-esteem among high school students (65). It utilizes the 4-points Likert scale ranging from 1 = Strongly Agree to 4 = Strongly Disagree. Regarding its reliability, the Rosenberg Self-Esteem Scale (RSES) demonstrates a Guttman Scale Coefficient of Reproducibility of 0.92, indicating excellent internal consistency. This scale's test-retest reliability over 2 weeks reveals correlations of 0.85 and 0.88, signifying excellent stability. A validation study on a Malay-translated version of the RSES revealed that, overall, the Malay version of the scale (m-RSES) is a valid and reliable tool with a Cronbach's alpha of 0.8 (66). For interpretation purposes, scores between 15 and 25 are interpreted as the respondents having a normal self-esteem range, while scores below 15 suggest low self-esteem. The Cronbach's alpha for our current study sample for RSES is 0.88.

### Statistical analysis

The collected data were keyed into the Statistical Package for Social Sciences (SPSS) software version 21. The completed Smartphone Addiction Scale (SAS), Social Interaction Anxiety Scale (SIAS), WHOQOL-BREF, and Rosenberg Self-Esteem Scale (RSES) questionnaires were scored according to their respective manuals or based on reviews of the literature. The calculated scores were then keyed into SPSS. Descriptive analyses were performed on the sociodemographic variables. Sociodemographic data, Social Interaction Anxiety Scale score, WHOQOL-BREF score, and the Rosenberg Self-esteem Scale score were treated as independent variables, while the Smartphone Addiction Scale score was treated as the dependent variable. Bivariate analysis was used to explore the relationship between the independent and dependent variables using the Fisher's exact test, Pearson Chi-Square analysis, and Pearson correlation coefficient. Furthermore, a multiple linear regression analysis was used to analyze the variables with a *p*-value of < 0.05 from the Pearson correlation coefficient test to control for confounders. Smartphone addiction was then analyzed as categorical data (using validated cut-off points) for comparison with sociodemographic data (using Fisher's exact test and Pearson chi-square test). On top of that, smartphone addiction's continuous data was used for correlation and regression analysis.

## Results

**Figure 1:**
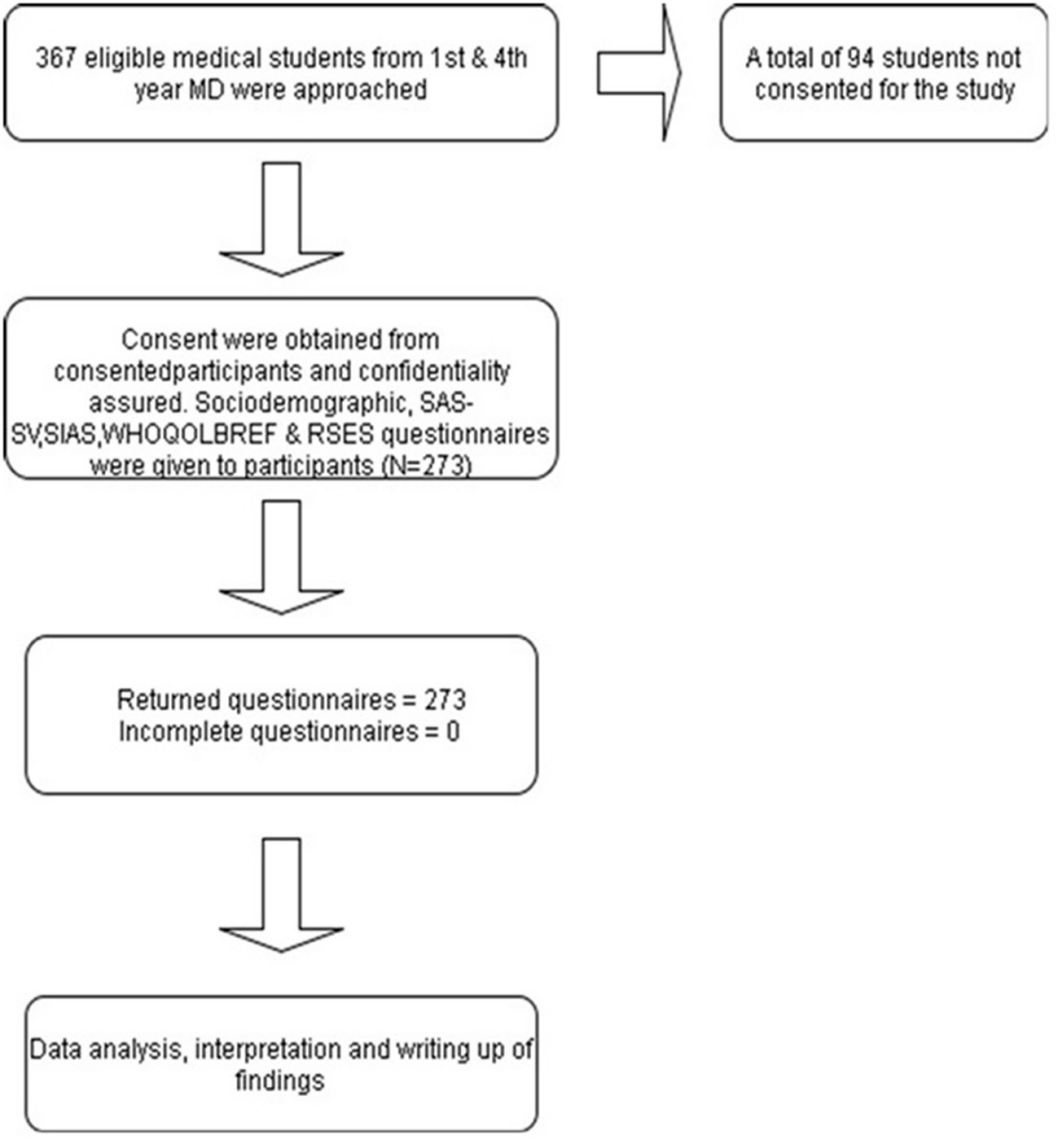
Flow diagram of study participants.

A total of 367 eligible medical students from year-1 and year-4 groups were approached to be recruited into this study as participants. However, 94 of them did not give their consent, thus excluding them from the final sample (Figure 1). The remaining students who consented were able to complete all questionnaires given to them. The total number of final respondents who participated and were included in the analysis was 273 students.

From the sociodemographic data, male students from both batches represent 30.7% (84 students) of the sample, while female students represent 69.2% (189 students). In terms of ethnicity, Malays made up most of the study sample, with 169 students (61.9%), followed by Indians (*n* = 48, 17.6%), Chinese (*n* = 44, 16.1%), and others (*n* = 12, 4.4%). The most predominant religion was Islam, with 184 followers (67.4%) followed sequentially by Buddhism (*n* = 38, 13.9%), Hinduism (*n* = 32, 11.7%), Christianity (*n* = 16, 5.8%), and others (*n* = 3, 1.1%). With regards to the place of origin, 214 students came from urban areas (78.4%) while the remaining 59 students (21.6%) came from rural areas. Moreover, regarding the parents' living status, a total of 25 students (9.16%) have lost their fathers, while three students (1.1%) have lost their mothers. In terms of parental marital status, 245 (89.7%) were in marriage, 10 (3.66%) were divorced, and 18 (6.6%) were widowed. Lastly, 31 students (11.4%) had an existing medical condition, and only 7 of them (2.56%) had an existing psychiatric condition or illness.

The rate of ESU among UKM medical students as measured using the SAS-SV was 48.4% (*n* = 132) (Table 1). From this, 32.6% of them (*n* = 43) are male and 67.4% (*n* = 89) are female. These figures were calculated based on the cutoff point of 31 score points for boys and 33 score points for girls (55).

**Table 1:** Sociodemographic information vs. excessive smartphone use.

		**No excessive smartphone use**		**Excessive smartphone use**		***p*-value**
		**Count**	**%**	**Count**	**%**	
Gender	Male	41	29.10%	43	32.60%	0.531^**^
	Female	100	70.90%	89	67.40%	
Ethnicity	Malay	81	57.40%	88	66.70%	0.388^*^
	Chinese	24	17.00%	20	15.20%	
	Indian	28	19.90%	20	15.20%	
	Others	8	5.70%	4	3.00%	
Religion	Islam	86	61.00%	98	74.20%	0.026^*^
	Buddha	21	14.90%	17	12.90%	
	Christian	13	9.20%	3	2.30%	
	Hindu	18	12.80%	14	10.60%	
	Others	3	2.10%	0	0.00%	
Year of MD	1st year	59	41.80%	65	49.20%	0.220^**^
	4th year	82	58.20%	67	50.80%	
Place of origin	Urban	107	75.90%	107	81.10%	0.229^**^
	Rural	34	24.10%	25	18.90%	
Father's living status	Yes	124	87.90%	124	93.90%	0.086^**^
	No	17	12.10%	8	6.10%	
Mother's living status	Yes	138	97.90%	132	100.00%	0.248^*^
	No	3	2.10%	0	0.00%	
Parents' marital status	Married	123	87.20%	122	92.40%	0.359^*^
	Divorced	6	4.30%	4	3.00%	
	Widowed	12	8.50%	6	4.50%	
Parents' household income	< RM1000	13	9.20%	13	9.80%	0.580^**^
	RM1000-RM4999	59	41.80%	46	34.80%	
	RM5000-RM10000	42	29.80%	40	30.30%	
	>RM10000	27	19.10%	33	25.00%	
Existing medical condition	Yes	13	9.20%	18	13.60%	0.250^**^
	No	128	90.80%	114	86.40%	
Existing psychiatric condition	Yes	3	2.10%	4	3.00%	0.715^*^
	No	138	97.90%	128	97.00%	

^*^Fisher exact test.

^**^Pearson chi-square. “Smartphone addition” and “No Excessive smartphone use” are categorized based on cut-off points of the SAS-SV (31 for males, and 33 for females).

Additionally, Fisher's exact test and Pearson Chi-Square were conducted to investigate whether there are statistically significant differences in sociodemographic information between the group of smartphone and non-smartphone addicts. The final result showed that there was a statistically significant difference in terms of religious beliefs between the smartphone addicts vis-à-vis the non-addicts with *p* = 0.026 (*p* < 0.05). The other factors do not seem to be different across the two above groups.

From the analysis, it may be determined that ESU was positively correlated with the domains of social anxiety (*r* = 0.173). This correlation was highly statistically significant (*p* = 0.004) (Table 2). Furthermore, ESU was negatively correlated with all domains of quality of life as measured by WHOQOL-BREF with *r* = −0.133 for physical health, *r* = −0.135 for psychological wellbeing, *r* = −0.232 for social relationships, and *r* = −0.260 for the environment. Moreover, the relationship was statistically significant (*p* < 0.05) for physical health and psychological wellbeing and was highly significant (*p* < 0.01) for social relationships and environment. On top of that, ESU was also found to exhibit a statistically significant negative correlation with self-esteem (*r* = −0.128).

**Table 2:** Correlations study excessive smartphone use vs. factors.

		**Excessive smartphone**	
		**use**	
		**Coefficient**	**Significant**
Social interaction anxiety scale (SIAS)	S.I.A.S Total	0.173^**^	0.004
WHOQOL - BREF	Physical health (Domain 1)	−0.133^*^	0.028
	Psychological wellbeing (Domain 2)	−0.135^*^	0.026
	Social relationships (Domain 3)	−0.232^**^	0.001
	Environment (Domain 4)	−0.260^**^	0.001
Rosenberg self-esteem scale (RSES)	R.S.E.S total	−0.128^*^	0.035

^*^Correlation is significant at the 0.05 level (2-tailed).

^**^Correlation is significant at the 0.01 level (2-tailed).

In Table 3, all the statistically significant variables from Table 2 were included in the regression analysis to estimate the relationship between the variables with ESU. In Table 4, the significant variable from sociodemographic data – religion – was incorporated into a model with all existing variables from Table 3 for further analysis. From results in Tables 3, 4, only WHOQOL-BREF (environment) & religion emerged as significant predictors of ESU out of independent variables that were computed in the regression model. The result from Table 3 showed that an increase of one point score in the WHOQOL-BREF environment domain will lead to a reduction in the ESU score by 0.248 (*p* = 0.009). Meanwhile, the results from Table 4 reveal that being Muslim is statistically significantly associated with an increased score for ESU by 0.595 (*p* = 0.027). And again, an increase of 1 point in the WHOQOL-BREF environment domain score is statistically significantly associated with the reduction of ESU score by 0.233 (*p* = 0.013) in Table 4.

**Table 3:** Regression analysis for relationship between variables and smartphone addiction.

**Model**	**Unstandardized coefficients**		**Standardized coefficients**	**t**	**Sig**.
	**B**	**Std. Error**	**Beta**		
S.I.A.S score	0.003	0.003	0.079	1.122	0.263
WHOQOL BREF (Physical health domain score)	0.000	0.004	−0.003	−0.034	0.973
WHOQOL BREF (Psychological wellbeing domain score)	0.005	0.004	0.114	1.242	0.215
WHOQOL BREF (Social relationships domain score)	−0.003	0.002	−0.123	−1.537	0.125
WHOQOL BREF (Environment domain score)	−0.011	0.004	−0.248	−2.626	0.009
Rosenberg self-esteem scale score	0.007	0.008	0.069	0.86	0.391

**Table 4:** Regression analysis for relationship between variables including religion with smartphone addiction.

**Model**	**Unstandardized coefficients**		**Standardized coefficients**	**t**	**Sig**.
	**B**	**Std. Error**	**Beta**		
S.I.A.S total	0.002	0.003	0.043	0.601	0.548
WHOQOL BREF (Physical health domain score)	−0.002	0.004	−0.045	−0.549	0.584
WHOQOL BREF (Psychological wellbeing domain score)	0.005	0.004	0.113	1.25	0.212
WHOQOL BREF (Social relationships domain score)	−0.004	0.002	−0.144	−1.808	0.072
WHOQOL BREF (Environment domain score)	−0.01	0.004	−0.233	−2.5	0.013
Rosenberg self-esteem scale score	0.007	0.008	0.066	0.826	0.410
Religion Islam	0.634	0.285	0.595	2.223	0.027
Religion Buddha	0.553	0.293	0.383	1.889	0.060
Religion Christian	0.312	0.306	0.146	1.02	0.309
Religion Hindu	0.51	0.293	0.328	1.743	0.083

## Discussion

This study was designed to identify the prevalence of ESU among UKM medical students and its relationship with social anxiety, quality of life, and self-esteem. Studies investigating the relationship between ESU and the above factors are still lacking, and to the best of our knowledge, have yet to be conducted in the local Malaysian context.

The rate of ESU in this study for both male and female students – as measured by the Short-version Smartphone Addiction Scale (SAS-SV) – is around 48%. This rate is similar to a local study (40.6%) (18). However, it is much higher than the rates recorded in similar studies using SAS-SV conducted in Korea, Switzerland, and universities in Iran, with 26.61% (14), 16.9% (17), and 9.3% (16) prevalence rates, respectively. A possible explanation for the high prevalence rate recorded in the current study is that the percentage of smartphone ownership in Malaysia is high (78% in 2018). Smartphones are also the most popular device for Malaysians to access the Internet (94.6%), according to statistics provided by the Malaysian Communications And Multimedia Commission (MCMC) in 2018 (67). The discrepancy between the findings in this study and two previous studies must be interpreted cautiously, due to the differences in terms of region and socio-cultural background of the studied populations.

In this study, the bivariate analysis yields a significant positive correlation between ESU with risk for social anxiety (*r* = 0.173, *p* = 0.004). This is consistent with findings from previous studies that demonstrated a higher strength of association between ESU and social anxiety (23, 26, 31). In the regression analysis, although the result was not statistically significant, there is a positive correlation between ESU and social anxiety. This non-statistically significant association in the regression analysis could be due to inadequate sample size. In other words, when other variables are factored into the equation, the relationship becomes weaker and non-significant. Another possible explanation is that perhaps medical students may have a lower threshold for social anxiety. Apart from a study that showed the prevalence rate of social anxiety among medical students can be as high as 59.5% (68), one cohort study conducted in a medical college in Turkey (with frequent follow-ups in 5-years intervals) revealed that the level of social anxiety among medical students reduces year-by-year, probably due to the positive effect of medical education (69). Three other studies also found that the prevalence of social anxiety among medical students is low (9.6, 18.7, and 21.8%, respectively) (70–72). In this study, the prevalence of social anxiety among medical students measured using SIAS is 20.14% (*n* = 55).

The relatively lower rate of anxiety among medical students compared to other students may be due to their higher psychological resilience. Psychological or mental resilience is defined as the ability to successfully and quickly cope with a crisis and to return to pre-crisis status (73). A study on the resilience between nursing and medical students has found that the level of resilience is higher among the latter (74). In the local context, the level of resilience among medical students in Malaysian public universities was reported to be moderately high, as measured by the Brief Resilience Scale (BRS) (75). A higher level of resilience has been associated with the reduction in risk for stress and anxiety as revealed in the study by Hjemdal et al. (*r* =−0.34, *p* < 0.001) and Rios-Risquez et al. (*r* =−0.62, *p* < 0.01) (75, 76).

Another possible explanation for the small association between ESU and social anxiety may be due to the different types of activities available on smartphones. People with social anxiety may prefer to engage in offline to online activities using smartphones. Elhai et al. reported that “non-social” features of smartphones are more related to anxiety as compared to “social” ones (50). This includes activities like news consumption, entertainment, and relaxation. This preference to use “non-social” content in smartphones is based on the Social Avoidance and Safety Behavior Theory (77–79).

There is a notion that excessive use or being addicted to a smartphone can negatively affect a person's quality of life (QoL). A part of the results of this study seems to support that view. Based on the bivariate (correlation) analysis, it appears that those with a higher risk for ESU will have a small but significant negative implication in several areas, such as physical & psychological health, social relationship, and the environment domains. In this study, excessive usage of smartphones has been shown to affect our physical health in several ways. This finding is similar to a recent study (43). Besides that, two studies showed that with an increase in time spent using smartphones or browsing the Internet, the rate or frequency of physical activity is significantly reduced (80, 81). Too much screening time using smartphones may also affect students' sleep duration and quality (82, 83). Moreover, physical issues such as neck disability may also occur among addicted smartphone users due to frequent neck flexion posture (84, 85).

Other parts of the psychological wellbeing constructs consist of positive and negative emotions and self-esteem, which may be jeopardized among smartphone addicts. Self-esteem has been shown to be directly and indirectly related to ESU (45, 48), a view that has been supported by the results of this study (Table 2). From the regression analysis (Tables 3, 4), the association between psychological wellbeing and self-esteem to ESU is negative. However, these associations are not statistically significant to be clinically meaningful. This can be attributed to the fact that in this sample of students, the purpose of smartphone usage is to enhance the students' profiles and obtain good feedback *via* social media (i.e., Facebook, and Instagram). Therefore, the usage of smartphones in this regard may boost students' self-esteem levels. This is largely similar to the findings made by Valkenburg et al. (86). Other factors such as peer relationships and a sense of belonging might explain the relationship between self-esteem and ESU. Past research reported that there is a mediating role played by self-esteem between student-student relationships and ESU (49).

As social beings, humans need to interact socially with others as part of their effort to maintain stable psychological wellbeing. Nowadays, people are more preoccupied with their phones instead of having the usual face-to-face social interaction. In this study, the correlation and regression analysis show that excessive use of a smartphone can impact social relationships in a negative way (Table 2: *r* = −0.232, *p* = 0.001, Table 4: β = −0.144, *p* = 0.072) albeit the relationship is not statistically significant. This is largely supported by other past studies. An experimental social study revealed that the presence of a smartphone negatively affects the quality of a conversation (87). In another study on a group of students (*n* = 768), the outcome demonstrated that the student-student relationship was negatively associated with ESU (49). A possible explanation for the non-significance of the social domain's regression analysis is the usage of smartphones in facilitating long-distance relationships. As the study participants were mostly staying in university-provided hostels, they may utilize video conferencing applications and platforms in their smartphones to preserve long-distance social interactions with their families.

Furthermore, the environment domain (as measured by WHOQOL-BREF) consistently displays a significant negative relationship with ESU across the different analyses conducted. Among the facets incorporated within this domain are financial status and home environment. Brown et al. found that the duration of smartphone usage among undergraduate students from low-income families was higher than those from higher-income families (88). This disparity may be due to a lack of other resources (i.e., PC or tablets) for low-income students to access the Internet. Prolonged usage of the Internet (*via* a smartphone) has been identified as one of the risk factors for ESU (89). Parents from families with lower economic status might not have the time and capacity to monitor their children's smartphone use (90). Another possible explanation for the significant negative relationship is respondents who are not satisfied with their living space or physical environment – for example, those living in a crowded home environment – might turn to ESU to escape from the uncomfortable living situation. Moreover, they may also do so to avoid reality by going into a virtual “ideal world”. For the home environment facet, a study discovered a significant positive correlation between daily work-home interference (WHI) and level of daily exhaustion, which is stronger for intensive smartphone users (z = 1.91, *p* < 0.05) (84, 91). WHI refers to the negative association between work and home domains (92).

Additionally, among the other results of this study is the significant association between an individual's belief system with the risk of ESU. This result needs to be interpreted cautiously as all religions – including Islam – generally promote healthy living. In this study, the sample students are predominantly Muslim, a fact that may influence the findings. In a report released by Pew Research Center, statistics show that the percentage of smartphone ownership has significantly increased in Muslim-majority countries like Lebanon and Jordan by 25–28% between 2015 to 2017 (93). A local study on Muslim university students has found that the majority of them are social media network users, which has affected their religious practice in daily life (94). With the abundance of religious information available, Malaysian Muslims spent nearly double the time accessing religious content online as compared to print media (95).

Looking at ESU from a slightly different angle, Parent et al. (19) suggested that researchers need to discuss the role of attachment dimensions to understand adults' relationship with their smartphones. According to Bowlby et al. (96), the attachment with an object is theorized to develop in tandem with the flourishing of the attachment dimension in infancy and remained throughout the life course. Studies looking at attachment styles among medical students showed around 48.8% (97) to 51.3% (98) students with secure attachment. Our current study does not investigate the different attachment styles of the participants. However, this provides an avenue for further research in this domain.

There are limitations to this study. Firstly, the sample size of 273 might not be enough for a good statistical analysis. Secondly, this study was conducted on a specific population (medical students), making the generalizability of the interpretation of its results to other populations to be limited. Furthermore, this study did not look into specific activities during the use of smartphones – like gaming, internet use, or social media. These activities represent another aspect to be explored in future studies. The small sample size and the sample heterogeneity in this study also did not allow Bonferroni corrections to be administered.

## Conclusion

This study offers insight into excessive smartphone use and its effects on Malaysian medical students. ESU is prevalent among UKM medical students regardless of their gender. Our study suggests that there is a significant positive correlation between ESU and social anxiety. We also discovered negative correlations between ESU and quality of life in various domains. Despite the limitations, we believe that this study may contribute to the development of new knowledge, particularly on the effects of smartphones on people's daily lives. Hopefully, this knowledge will help to guide users to maximize the potential of a smartphone to enrich their lives while averting the pitfalls. Further studies on smartphone use are necessary to explore how and under what conditions would smartphone use – despite being excessive – still be beneficial to users. The studies may also explore what other conditions would smartphone use be considered harmful.

## Data availability statement

The original contributions presented in the study are included in the article/supplementary material, further inquiries can be directed to the corresponding author/s.

## Ethics statement

The studies involving human participants were reviewed and approved by Research and Ethics Committee, Faculty of Medicine, Universiti Kebangsaan Malaysia (Project Code: FF-2018-243). The patients/participants provided their written informed consent to participate in this study.

## Author contributions

MM, SS, and NN contributed to conception and design of the study. AA, KS, NB, NM, and SK collected and organized the database. MN performed the statistical analysis. HM, AA, KS, NB, NM, SK, MM, NN, and SS wrote sections of the manuscript. All authors contributed to manuscript revision, read, and approved the submitted version.

## Conflict of interest

The authors declare that the research was conducted in the absence of any commercial or financial relationships that could be construed as a potential conflict of interest.

## Publisher's note

All claims expressed in this article are solely those of the authors and do not necessarily represent those of their affiliated organizations, or those of the publisher, the editors and the reviewers. Any product that may be evaluated in this article, or claim that may be made by its manufacturer, is not guaranteed or endorsed by the publisher.

## References

[1] Department of statistics Malaysia D. ICT Use and Access By Individuals and Households Survey Report, Malaysia, 2021. Department of statistics Malaysia; (2022) (accessed April 28, 2022).

[2] Stawarz K Preist C Coyle D. Use of smartphone apps, social media, and web-based resources to support mental health and wellbeing: Online survey. JMIR Mental Health. (2019) 6:e12546. 10.2196/12546 31301126 PMC6659390

[3] Lüdtke T Pult LK Schröder J Moritz S Bücker L A. randomized controlled trial on a smartphone self-help application (Be Good to Yourself) to reduce depressive symptoms. Psychiatry Res. (2018) 269:753–62. 10.1016/j.psychres.2018.08.113 30273901

[4] Linardon J Cuijpers P Carlbring P Messer M Fuller-Tyszkiewicz M. The efficacy of app-supported smartphone interventions for mental health problems: a meta-analysis of randomized controlled trials. World Psychiatry. (2019) 18:325–36. 10.1002/wps.20673 31496095 PMC6732686

[5] Metelmann C Metelmann B Schuffert L Hahnenkamp K Vollmer M Brinkrolf P. Smartphone apps to support laypersons in bystander CPR are of ambivalent benefit: a controlled trial using medical simulation. Scand J Trauma Resusc Emerg Med. (2021) 29:76. 10.1186/s13049-021-00893-3 34082804 PMC8173850

[6] Abbott LM Smith SD. Smartphone apps for skin cancer diagnosis: Implications for patients and practitioners. Austral J Dermatol. (2018) 59:168–70. 10.1111/ajd.12758 29292506

[7] Khan A Bibi F Dilshad M Ahmed S Ullah Z Ali H. Accident detection and smart rescue system using Android smartphone with real-time location tracking. Int J Adv Comput Sci Applic. (2018) 9:341–55. 10.14569/IJACSA.2018.090648

[8] Organization WH. ICD-11 for mortality and morbidity statistics (2018).

[9] American Psychiatric Association D Association AP. Diagnostic and statistical manual of mental disorders: DSM-5. Washington, DC: American psychiatric association. (2013). 10.1176/appi.books.9780890425596

[10] Yu S Sussman S. Does smartphone addiction fall on a continuum of addictive behaviors? Int J Environ Res Public Health. (2020) 17:422. 10.3390/ijerph17020422 31936316 PMC7014405

[11] Lin Y-H Chang L-R Lee Y-H Tseng H-W Kuo TBJ Chen S-H. Development and Validation of the Smartphone Addiction Inventory (SPAI). PLoS ONE. (2014) 9:e98312. 10.1371/journal.pone.0098312 24896252 PMC4045675

[12] Kuss DJ Billieux J. Technological addictions: Conceptualisation, measurement, etiology and treatment. Addict Behav. (2017) 64:231–3. 10.1016/j.addbeh.2016.04.005 27136694

[13] Griffiths M. Technological addictions. In: Clinical Psychology Forum. Division of C linical Psychology of the British Psychol Soc. (1995).

[14] Lee J Sung M-J Song S-H Lee Y-M Lee J-J Cho S-M. Psychological factors associated with smartphone addiction in South Korean adolescents. J Early Adolesc. (2018) 38:288–302. 10.1177/0272431616670751

[15] Choksi ST Patel N A. study to find out the correlation of mobile phone addiction with anxiety, depression, stress and sleep quality in the college students of Surat city. Int J Curr Res Rev. (2021) 13:137–42. 10.31782/IJCRR.2021.13812

[16] Yahyazadeh S Fallahi-Khoshknab M Norouzi K Dalvandi A. The prevalence of smart phone addiction among students in medical sciences universities in Tehran 2016. Adv Nurs Midwifery. (2017) 26:1–10. Available online at: https://journals.sbmu.ac.ir/en-jnm/article/view/15723

[17] Haug S Castro RP Kwon M Filler A Kowatsch T Schaub MP. Smartphone use and smartphone addiction among young people in Switzerland. J Behav Addict. (2015) 4:299–307. 10.1556/2006.4.2015.037 26690625 PMC4712764

[18] Lei LY-C Ismail MA-A Mohammad JA-M Yusoff MSB. The relationship of smartphone addiction with psychological distress and neuroticism among university medical students. BMC Psychol. (2020) 8:97. 10.1186/s40359-020-00466-6 32917268 PMC7488412

[19] Parent N Shapka J. Moving beyond addiction: An attachment theory framework for understanding young adults' relationships with their smartphones. Hum Behav Emerg Technol. (2020) 2:179–85. 10.1002/hbe2.180

[20] Bowlby J. Separation of mother and child. Lancet. (1958) 271:480. 10.1016/S0140-6736(58)90794-3

[21] King ALS Valença AM Silva ACO Baczynski T Carvalho MR Nardi AE. Nomophobia: Dependency on virtual environments or social phobia? Comput Human Behav. (2013) 29:140–4. 10.1016/j.chb.2012.07.025

[22] Konok V Pogány Á Miklósi Á. Mobile attachment: Separation from the mobile phone induces physiological and behavioural stress and attentional bias to separation-related stimuli. Comput Human Behav. (2017) 71:228–39. 10.1016/j.chb.2017.02.002

[23] Jin Jeong Y Suh B Gweon G. Is smartphone addiction different from Internet addiction? comparison of addiction-risk factors among adolescents. Behav Inf Technol. (2020) 39:578–93. 10.1080/0144929X.2019.1604805

[24] Basti BD Udayar SE Kumar JP Boyapati HNB. Association of anxiety and depression in relation to smartphone addiction among university students in. India. Malaysian J Public Health Med. (2021) 21:71–8. Available online at: 10.37268/mjphm/vol.21/no.3/art.943

[25] Wan Ismail WS Sim ST Tan K-A Bahar N Ibrahim N Mahadevan R. The relations of internet and smartphone addictions to depression, anxiety, stress, and suicidality among public university students in Klang Valley, Malaysia. Perspect Psychiatr Care. (2020) 56:949–55. 10.1111/ppc.12517 32363648

[26] Aroyewun FT Osinowo HO. Depression, stress and obsessive–compulsive disorder as predictors of smartphone addiction among smartphone users in Ibadan. African J Psychol Stud Soc Issues. (2019) 22:80–7. Available online at: https://www.ajol.info/index.php/ajpssi/article/view/218420

[27] Kim S-G Park J Kim H-T Pan Z Lee Y McIntyre RS. The relationship between smartphone addiction and symptoms of depression, anxiety, and attention-deficit/hyperactivity in South Korean adolescents. Ann Gen Psychiatry. (2019) 18:1–8. 10.1186/s12991-019-0224-8 30899316 PMC6408841

[28] Telgote SA Ghogare AS Khadse V Karwande SG. Smartphone Addiction and its Impact on Insomnia among the Undergraduate Medical Students of a Teaching Hospital of Maharashtra, India-A Cross-sectional study. J Clin. Diagn. Res. (2021) 15:5. 10.7860/JCDR/2021/52819.15753

[29] Enez Darcin A Kose S Noyan CO Nurmedov S Yilmaz O Dilbaz N. Smartphone addiction and its relationship with social anxiety and loneliness. Behav Inf Technol. (2016) 35:520–5. 10.1080/0144929X.2016.1158319

[30] Pugh S. Investigating the relationship between smartphone addiction, social anxiety, self-esteem, age & gender. (2017).

[31] Leigh E Clark DM. Understanding Social Anxiety Disorder in Adolescents and Improving Treatment Outcomes: Applying the Cognitive Model of Clark and Wells (1995). Clin Child Fam Psychol Rev. (2018) 21:388–414. 10.1007/s10567-018-0258-5 29654442 PMC6447508

[32] Ithnain N Ghazali SE Jaafar N. Relationship between smartphone addiction with anxiety and depression among undergraduate students in Malaysia. Int J Health Sci Res. (2018) 8:163–71.

[33] Elhai JD Tiamiyu M Weeks J. Depression and social anxiety in relation to problematic smartphone use: The prominent role of rumination. Internet Res. (2018) 28:315–32. 10.1108/IntR-01-2017-0019

[34] TraÅÝ Z. Internet addiction and loneliness as predictors of internet gaming disorder in adolescents. Educ Res Rev. (2019) 14:465–73. 10.5897/ERR2019.376832612421

[35] Fu X Liu J Liu RD Ding Y Wang J Zhen R. Parental monitoring and adolescent problematic mobile phone use: the mediating role of escape motivation and the moderating role of shyness. Int J Environ Res Public Health. (2020) 17:1487. 10.3390/ijerph17051487 32106623 PMC7084728

[36] Li J Zhan D Zhou Y Gao X. Loneliness and problematic mobile phone use among adolescents during the COVID-19 pandemic: The roles of escape motivation and self-control. Addict Behav. (2021) 118:106857. 10.1016/j.addbeh.2021.106857 33676160 PMC8598166

[37] Jaafar NRN Bahar N Ibrahim N Wan Ismail WS Baharudin A. Excessive internet use in young women: What are the implications? Curr Opin Psychiatry. (2017) 30:260–7. 10.1097/YCO.0000000000000336 28426547

[38] Lee Y-K Chang C-T Cheng Z-H Lin Y. How social anxiety and reduced self-efficacy induce smartphone addiction in materialistic people. Soc Sci Comput Rev. (2018) 36:36–56. 10.1177/0894439316685540

[39] Munderia R Singh R editors. The mediating effect of smartphone addiction on the relationship between social skills and psychological well-being. In: International Conference on Human-Computer Interaction, Springer (2021). 10.1007/978-3-030-78645-8_46

[40] You Z Zhang Y Zhang L Xu Y Chen X. How does self-esteem affect mobile phone addiction? The mediating role of social anxiety and interpersonal sensitivity. Psychiat Res. (2019) 271:526–31. 10.1016/j.psychres.2018.12.040 30553099

[41] Zsido AN Arato N Lang A Labadi B Stecina D Bandi SA. The role of maladaptive cognitive emotion regulation strategies and social anxiety in problematic smartphone and social media use. Pers Individ Dif. (2021) 173:110647. 10.1016/j.paid.2021.110647

[42] Kim S. World Health Organization quality of life (WHOQOL) assessment. In: Encyclopedia of quality of life and wellbeing research, eds. AC Michalos, Dordrecht: Springer Netherlands. (2020). p. 1–2. 10.1007/978-3-319-69909-7_3282-2

[43] Shahrestanaki E Maajani K Safarpour M Ghahremanlou HH Tiyuri A Sahebkar M. The relationship between smartphone addiction and quality of life among students at Tehran University of medical sciences. Addicta. (2020) 7:23–32. 10.15805/addicta.2020.7.1.008032431237

[44] Buctot DB Kim N Kim JJ. Factors associated with smartphone addiction prevalence and its predictive capacity for health-related quality of life among Filipino adolescents. Child Youth Serv Rev. (2020) 110:104758. 10.1016/j.childyouth.2020.104758

[45] Andreassen CS Pallesen S Griffiths MD. The relationship between addictive use of social media, narcissism, and self-esteem: Findings from a large national survey. Addict Behav. (2017) 64:287–93. 10.1016/j.addbeh.2016.03.006 27072491

[46] Kim E Koh E. Avoidant attachment and smartphone addiction in college students: The mediating effects of anxiety and self-esteem. Comput Human Behav. (2018) 84:264–71. 10.1016/j.chb.2018.02.037

[47] Mohamed SM Mostafa MH. Impact of smartphone addiction on depression and self-esteem among nursing students. Nursing Open. (2020) 7:1346–53. 10.1002/nop2.506 32802355 PMC7424452

[48] Samaha M Hawi NS. Relationships among smartphone addiction, stress, academic performance, and satisfaction with life. Comput Human Behav. (2016) 57:321–5. 10.1016/j.chb.2015.12.045

[49] Wang P Zhao M Wang X Xie X Wang Y Lei L. Peer relationship and adolescent smartphone addiction: The mediating role of self-esteem and the moderating role of the need to belong. J Behav Addict. (2017) 6:708–17. 10.1556/2006.6.2017.079 29254360 PMC6034960

[50] Elhai JD Levine JC Dvorak RD Hall BJ. Non-social features of smartphone use are most related to depression, anxiety and problematic smartphone use. Comput Human Behav. (2017) 69:75–82. 10.1016/j.chb.2016.12.023

[51] Chandra A Nongkynrih B Gupta SK. Role of smartphone technology in medical education in India. Indian J Commun Family Med. (2019) 5:103. 10.4103/IJCFM.IJCFM_42_19

[52] Tamil M. Calculate Your Own Sample Size. Kuala Lumpur (MY): Department of Community Health and Sekretariat of Medical Research and Industry, University Kebangsaan Malaysia Medical Centre. (2008).

[53] Kwon M Kim D-J Cho H Yang S. The smartphone addiction scale: development and validation of a short version for adolescents. PLoS ONE. (2013) 8:e83558. 10.1371/journal.pone.0083558 24391787 PMC3877074

[54] Mattick RP Clarke JC. Development and validation of measures of social phobia scrutiny fear and social interaction anxiety. Behav Res Ther. (1998) 36:455–70. 10.1016/S0005-7967(97)10031-6 9670605

[55] Kwon M Lee J-Y Won W-Y Park J-W Min J-A Hahn C. Development and validation of a smartphone addiction scale (SAS). PLoS ONE. (2013) 8:e56936. 10.1371/journal.pone.0056936 23468893 PMC3584150

[56] Ching SM Yee A Ramachandran V Sazlly Lim SM Wan Sulaiman WA Foo YL. Validation of a Malay version of the smartphone addiction scale among medical students in Malaysia. PLoS ONE. (2015) 10:e0139337. 10.1371/journal.pone.0139337 26431511 PMC4592235

[57] Peters L. Discriminant validity of the social phobia and anxiety inventory (SPAI), the social phobia scale (SPS) and the social interaction anxiety scale (SIAS). Behav Res Ther. (2000) 38:943–50. 10.1016/S0005-7967(99)00131-X 10957828

[58] Ke GN Wong SF A. healthy mind for problematic internet use. Cyberpsychol, Behav Soc Netw. (2018) 21:637–45. 10.1089/cyber.2018.0072 30256674

[59] Raj SLY Yasin MAM Othman Z Othman A. Drinking motives as mediator between social anxiety and alcohol use among private university students in Klang Valley. Proc Soc Behav Sci. (2016) 219:614–9. 10.1016/j.sbspro.2016.05.041

[60] World Health Organisation W. WHOQOL-BREF Introduction, administration, scoring and generic version of the assessment (1996). Available online at: https://www.who.int/mental_health/media/en/76.pdf (accessed March 21, 2022).

[61] Bonomi AE Patrick DL Bushnell DM Martin M. Validation of the United States' version of the world health organization quality of life (WHOQOL) instrument. J Clin Epidemiol. (2000) 53:1–12. 10.1016/S0895-4356(99)00123-7 10693897

[62] Group W. Development of the World Health Organization WHOQOL-BREF quality of life assessment. Psychol Med. (1998) 28:551–8. 10.1017/S0033291798006667 9626712

[63] Hawthorne G Herrman H Murphy B. Interpreting the WHOQOL-BREF: Preliminary population norms and effect sizes. Soc Indic Res. (2006) 77:37–59. 10.1007/s11205-005-5552-1

[64] Hasanah C Naing L Rahman A. World Health Organization quality of life assessment: brief version in Bahasa Malaysia. Medical J Malaysia. (2003) 58:79–88. Available online at: https://pubmed.ncbi.nlm.nih.gov/14556329/14556329

[65] Rosenberg M. Rosenberg self-esteem scale (RSE). Acceptance and commitment therapy. Measures Package. (1965) 61:18. 10.1037/t01038-000

[66] Jamil M. Validity and reliability study of Rosenberg self-esteem scale in Seremban school children. Malaysian J Psychiatry. (2006) 15:35–9. Available online at: https://www.academia.edu/3345857/Validity_and_reliability_study_of_Rosenberg_Self_Esteem_Scale_in_Seremban_school_children

[67] Malaysian Communications and Multimedia Communication M. Hand phone user survey (2018). Available online at: https://www.mcmc.gov.my/skmmgovmy/media/General/pdf/HPUS2018.pdf (accessed March 21, 2022).

[68] Elhadad AA Alzaala MA Alghamdi RS Asiri SA Algarni AA Elthabet MM. Social phobia among Saudi medical students. Middle East Curr Psychiat. (2017) 24:68–71. 10.1097/01.XME.0000513066.80386.b6

[69] S. Ak CK. Social anxiety in medical students: A five-year follow-up. Eur Psychiat. (2016) 33S:S389. 10.1016/j.eurpsy.2016.01.1108

[70] Izgiç F Akyüz G Dogan O Kugu N. Social phobia among university students and its relation to self-esteem and body image. Canad J Psychiat. (2004) 49:630–4. 10.1177/070674370404900910 15503736

[71] Amit J. Jogdande AG. Study of Social anxiety disorder in medical students in an urban area. Int J Sci Res. (2017) 6:4329. Avaialble online at: 10.36106/ijsr

[72] Latifeh Y Aless A Salloum R. The prevalence of social phobia and its potential demographics characteristics risk factors, among medical students in Syrian Private University. (2018).

[73] De Terte I Stephens C. Psychological resilience of workers in high-risk occupations. (2014). 10.1002/smi.2627 25476960

[74] Zhao F Guo Y Suhonen R Leino-Kilpi H. Subjective wellbeing and its association with peer caring and resilience among nursing vs medical students: A questionnaire study. Nurse Educ Today. (2016) 37:108–13. 10.1016/j.nedt.2015.11.019 26694789

[75] Hjemdal O Vogel PA Solem S Hagen K Stiles TC. The relationship between resilience and levels of anxiety, depression, and obsessive–compulsive symptoms in adolescents. Clin Psychol Psychother. (2011) 18:314–21. 10.1002/cpp.719 20806419

[76] Ríos-Risquez MI García-Izquierdo M Sabuco-Tebar EdlA Carrillo-Garcia C Martinez-Roche ME. An exploratory study of the relationship between resilience, academic burnout and psychological health in nursing students. Contemp Nurse. (2016) 52:430–9. 10.1080/10376178.2016.1213648 27436758

[77] Kashdan TB. Social anxiety spectrum and diminished positive experiences: Theoretical synthesis and meta-analysis. Clin Psychol Rev. (2007) 27:348–65. 10.1016/j.cpr.2006.12.003 17222490

[78] Rachman S Radomsky AS Shafran R. Safety behaviour: A reconsideration. Behav Res Ther. (2008) 46:163–73. 10.1016/j.brat.2007.11.008 18199423

[79] Powers MB Smits JA Telch MJ. Disentangling the effects of safety-behavior utilization and safety-behavior availability during exposure-based treatment: a placebo-controlled trial. J Consult Clin Psychol. (2004) 72:448. 10.1037/0022-006X.72.3.448 15279528

[80] Kim S-E Kim J-W Jee Y-S. Relationship between smartphone addiction and physical activity in Chinese international students in Korea. J Behav Addict. (2015) 4:200–5. 10.1556/2006.4.2015.028 26551911 PMC4627682

[81] Männikkö N Billieux J Kääriäinen M. Problematic digital gaming behavior and its relation to the psychological, social and physical health of Finnish adolescents and young adults. J Behav Addict. (2015) 4:281–8. 10.1556/2006.4.2015.040 26690623 PMC4712762

[82] Demirci K Akgönül M Akpinar A. Relationship of smartphone use severity with sleep quality, depression, and anxiety in university students. J Behav Addict. (2015) 4:85–92. 10.1556/2006.4.2015.010 26132913 PMC4500888

[83] Soni R Upadhyay R Jain M. Prevalence of smart phone addiction, sleep quality and associated behaviour problems in adolescents. Int J Res Med Sci. (2017) 5:515–9. 10.18203/2320-6012.ijrms2017014236371611

[84] Mustafaoglu R Yasaci Z Zirek E Griffiths MD Ozdincler AR. The relationship between smartphone addiction and musculoskeletal pain prevalence among young population: a cross-sectional study. Korean J Pain. (2021) 34:72–81. 10.3344/kjp.2021.34.1.72 33380570 PMC7783853

[85] Alsalameh AM Harisi MJ Alduayji MA Almutham AA Mahmood FM. Evaluating the relationship between smartphone addiction/overuse and musculoskeletal pain among medical students at Qassim University. J Family Med Prim Care. (2019) 8:2953–9. 10.4103/jfmpc.jfmpc_665_19 31681674 PMC6820402

[86] Valkenburg PM Peter J Schouten AP. Friend networking sites and their relationship to adolescents' wellbeing and social self-esteem. CyberPsychol Behav. (2006) 9:584–90. 10.1089/cpb.2006.9.584 17034326

[87] Abeele MMV Antheunis ML Schouten AP. The effect of mobile messaging during a conversation on impression formation and interaction quality. Comput Human Behav. (2016) 62:562–9. 10.1016/j.chb.2016.04.005

[88] Brown K Campbell SW Ling R. Mobile Phones Bridging the Digital Divide for Teens in the US? Future Internet. (2011) 3:144–58. 10.3390/fi3020144

[89] Choi SW Kim DJ Choi JS Ahn H Choi EJ Song WY. Comparison of risk and protective factors associated with smartphone addiction and Internet addiction. J Behav Addict. (2015) 4:308–14. 10.1556/2006.4.2015.043 26690626 PMC4712765

[90] Lee J Lim H Allen J Choi G Jung J. Smartphone addiction and depression among low-income boys since COVID-19: The moderating effect of being an only child. Healthcare. (2021) 9:1350. 10.3390/healthcare9101350 34683030 PMC8544461

[91] Derks D Bakker AB. Smartphone use, work–home interference, and burnout: a diary study on the role of recovery. Appl Psychol. (2014) 63:411–40. 10.1111/j.1464-0597.2012.00530.x

[92] Van Hooff ML Geurts SA Kompier MA Taris TW. Work–home interference: How does it manifest itself from day to day? Work Stress. (2006) 20:145–62. 10.1080/02678370600915940

[93] Poushter J Bishop C Chwe H. Social media use continues to rise in developing countries but plateaus across developed ones. Pew Res Center. (2018) 22:2–19. Available online at: https://www.pewresearch.org/global/2018/06/19/social-media-use-continues-to-rise-in-developing-countries-but-plateaus-across-developed-ones/

[94] Sharaf GM Musa MA Rahman AA. An examination of social networking sites usage among muslims student in islamic perspectives. Int J Eng Adv Technol. (2012) 1:273–8. Available online at: https://eprints.lancs.ac.uk/id/eprint/58646/

[95] Mahadi MA. A case study of religious engagement online: How Malaysian Muslim students access Islamic information. Rochester Institute of Technology. (2013).

[96] Bowlby J. Attachment and Loss: Volume I: Attachment. In: International Psycho-Analytical Library. (1969). vol. 79. p. 1–401.

[97] Atherton K Chisholm A Rutter L Peters S Fletcher I. Breaking barriers in clinical communication: are securely attached doctors more empathetic doctors? Reinvention. (2009) 2:2–6. Available online at: https://eprints.lancs.ac.uk/id/eprint/58646

[98] Hick RF. Does medical students' attachment style affect their ability to communicate with patients in emotional distress? University of Liverpool. (2009).

